# Changes in character strengths after watching movies: when to use rasch analysis

**DOI:** 10.1186/s13104-020-05424-4

**Published:** 2021-01-06

**Authors:** Sirinut Siritikul, Sirikorn Chalanunt, Chitipat Utrapiromsook, Suchanard Mungara, Tinakon Wongpakaran, Nahathai Wongpakaran, Pimolpun Kuntawong, Danny Wedding

**Affiliations:** 1 Faculty of Medicine, Chiang Mai University, Chiang Mai, Thailand; 2 Psychotherapy unit, Department of Psychiatry, Faculty of Medicine, Chiang Mai University, 110 Intawaroros Rd., T. Sriphum, A. Muang, Chiang Mai, Thailand; 3 Saybrook University, Oakland, California USA

**Keywords:** Movies, Character strength, Composite scale, Prudence, Humility, Self-regulation, Gratitude, Medical students

## Abstract

**Objective:**

Professionalism is a critical part of a medical education, and various activities have been proposed to enhance professionalism among medical students. Watching films is an activity to promote character related to professionalism. Limitation of such is a single group pre-posttest design raising concerns about the errors of measurement. The study aimed to demonstrate a method to deal with this design using Rasch analysis.

**Results:**

This study used a pre-posttest design with 40 first year medical students. All participated in a 3-day activity that involved watching four selected movies: *Twilight, Gandhi*, *The Shawshank Redemption* and *Amélie.* These films offer compelling illustrations of the themes of self-regulation, humility, prudence and gratitude, respectively. All participants completed a 10-item composite scale (PHuSeG) addressing these themes before and after watching the movies. When determining who benefitted from the intervention, paired t-tests on the results of a Rasch analysis were used to evaluate changes between pre- and posttest. Using Rasch analyses, we could document the stability of the items from pre- to posttest, and significant changes at both the individual and group levels, which is a useful and practical approach for pre- and posttest design. Moreover, it helps validate the psychometric property of the instrument used.

## Introduction

Medicine is a demanding profession. In addition to knowledge, medical students are expected to be skilled at relating to patients, the patients’ caregivers and other healthcare professionals [1, 2]. The core attributes pertinent to human connection are found across all cultures. Such attributes include the ability to build a therapeutic relationship with patients, skills in providing patient-centered care, effective communication and interpersonal skills [3, 4].

Character strengths and virtues are considered core characteristics valued by moral philosophers and religious thinkers [5]. The virtues associated with positive psychology are wisdom and knowledge, courage, humility, justice, temperance and transcendence [6]. Several character strengths related to the medical professionalism include medical ethics. Studies have shown that some clinical dilemmas require the character strengths of honesty, wisdom, prudence, kindness, courage, hope and wisdom to guide ethical decision-making [7, 8]. Culturally, the characters or virtues associated with being a good doctor include self-regulation, prudence, humility and gratitude [1]. These characteristics are briefly described below.

*Self-regulation* includes behaviors such as calmness and patience. For medical students, self-regulation involves maintaining competence such as taking appropriate action to prevent conflicts of interest when dealing with a pharmaceutical company [9].

*Prudence* is a strength described as being careful about one’s choices, such as not taking undue risks [10]. This value is commonly found among physicians who are noted for their clinical judgment. One topical and salient example of where empathy and prudence are especially needed is finding strategies and resources to improve quality of care and to ease the anxiety concerning nurses caring for patients with COVID‐19 [11].

*Humility* is a character strength characterized by humility and freedom from arrogance. This value is related to work success [12], as well as psychological strength and effectiveness [13].

The last strength is *gratitude,* which is characterized by a general state of thankfulness; gratitude is defined as “the appreciation of what is valuable and meaningful to oneself” [14].

A number of strategies are available to promote character strengths such as mindfulness-based training programs and digital-free tourism [15, 16]; one activity involves watching movies. Cinema-education [17] and related interventions using films attempt to promote psychological health [18]. Research has shown that students’ positive orientation and growth initiative can be promoted using a systematic movie-based teaching course [19, 20].

To measure the changes resulting from watching movies, we usually use a pre-post design. This design is used in both academic and in clinical settings. In a pedagogic setting, medical educators may be interested in how students change after a class or intervention. This pre-post evaluation can; however, be biased because of its ordinality. Rasch analysis is one way of addressing this limitation because data have been transformed onto an interval scale. In Rasch analysis, both item difficulty and person ability and parameters are considered and plotted on the same interval-level scale, to reduce errors of measurement [21]. Rather than only examining the change at the group level, the Rasch model analyzes change at an individual level. Rasch analysis allows us to have interval scores for each person, making the comparison between pre- and posttest more accurate.

The aim of the study was to demonstrate the advantage of using Rasch analysis in one group employing a pre-post design to allow (1) verification of the stability of the items, (2) assessment of who will or will not benefit from this particular intervention and (3) assessment of the reliability of the measurement.

## Main text

### Methods

This research was approved by the ethics committee of the Faculty of Medicine, Chiang Mai University, Thailand.

### Participants and procedure

Forty students joined the movie project as part of the extracurricular activity of the general education curriculum. The subjects comprised 40 medical students: 25 males and 15 females between 19 and 21 years old. Before watching the films, each participant completed a composite scale determining character strength. On the first day, the participants watched two movies, i.e., *The Shawshank Redemption* and *Twilight*. After viewing each movie, a group of five participants discussed and shared their opinions about the movies. Then all participants wrote down their own summary about their attitudes toward each movie. On the second day, the same process was repeated with the other two movies, i.e., *Gandhi* and *Amélie*. Then after viewing all four movies, all participants completed the same questionnaires (Additional file 1: Figure S1).

### Measurement

The PHuSeG scale constitutes a composite scale measuring prudence, humility, self-regulation and gratitude [22]. The scale measures positive psychology character strengths associated with professionalism. The PHuSeG consists of ten items and five rating responses. In our previous study of construct validity using the Rasch measurement model, the scale was shown to be unidimensional, and all items had mean square fit statistics between 0.76 and 1.37, which fell within the recommended range of 0.5 to 1.5 [23], with good person and item reliability (0.80 and 0.91, respectively). Cronbach’s alpha was 0.84. Three items assessed self-regulation, two assessed humility, four items were selected to measure prudence and one item measured gratitude. The summed raw scores of the PHuSeG scale range from 10 to 50. The higher the score, the greater the positive character strength is present. The study sample yielded a Cronbach’s alpha of 0.81 for pretest and 0.84 for posttest.

### Films

Four films were used to illustrate each positive attribute based on expert recommendations in the book entitled, “*Positive psychology at the movies: using films to build virtues and character strengths”* [24]. The authors selected the following films based on these criteria: *Gandhi* (Richard Attenborough, 1982), demonstrating humility; *Twilight* (Catherine Hardwicke, 2008), demonstrating self-regulation; *The Shawshank Redemption* (Frank Darabont, 1994), demonstrating prudence and *Amelie* (Jean-Pierre Jeunet, 2001), demonstrating gratitude.

### Data analysis

Descriptive analyses were conducted on sociodemographics. To compare differences of all dependent variables after intervention, paired t-tests were used. Both PHuSeG ordinal (raw) and interval (Rasch) scores were analyzed separately and compared. For all the analyses, levels of significance were set at P < 0.05 and IBM SPSS, Version 22 was used for all analyses.

Rasch models were adopted for this pre-post design analysis because they provided information at individual levels, allowing us to pinpoint who benefits from the intervention. For Rasch analyses, when the data fit the model, interval measures are collected from ordinal scores, yielding more accurate measures of change. Individuals or subjects can be measured within a common frame of reference covering different time points so that the measurement of change becomes a precise numerical representation on a shared linear scale in additive measurement units (logits). Rasch analysis also ensures the invariance (stability) of the instrument across time points. According to Wright [25], the Rasch model can provide answers for two different research questions: one focuses on changes in student performance; the other focuses on changes in item difficulty over time. Before any interpretations, examining the fit of the data to the model is required. Fit statistics along with a principal component analysis (PCA) can determine whether the assumption empirically supports unidimensionality [26].

For fit statistics, outlier-sensitive fit statistics mean square (OUTFIT.MnSq) and information-weighted fit statistics mean square (INFIT.MnSq) ranged from 0.5 to 1.5; these values are considered acceptable [23]. The separation and reliability for the persons and items were examined laying the support for the validity of interpretations. Acceptable values for separation cutoff, person reliability, and item reliability, provided by Wright and Stone [27], were ≥ 2, ≥ 0.70, and ≥ 0.8, respectively. To test for item stability for pre-post comparisons, the differential item function (DIF) was evaluated. The significant DIF was considered when DIF contrast was ≥ 0.64 [28]. Winsteps, Version 4.7.0 was employed for Rasch analysis.

## Results

Items with differential item functioning for pre-post comparisons were evaluated, and no significant DIF was observed (Additional file 2: Table S1).

**Figure 1:**
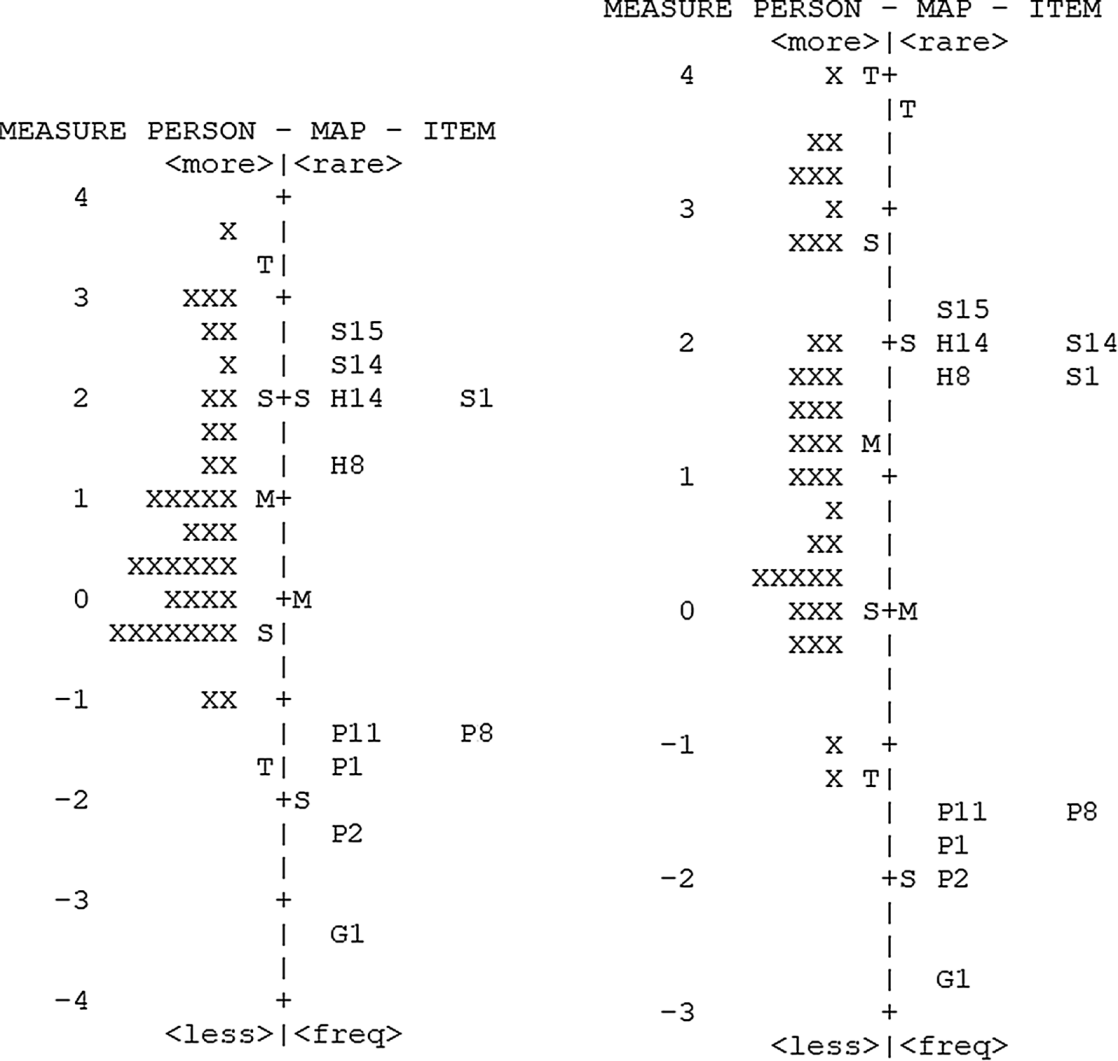
Item-Person Wright Map comparing pretest and posttest. X (on the left side of the vertical dash line)  = one person, Alphabet and number (on the right side of vertical dash line)  = items from composite scale, M = mean, S = 1 standard deviation, T = 2 1 standard deviation

Figure 1 compares the PhuSeG scale items being measured at pre- and posttest. Notably, almost all items maintain their location at the variable, denoting invariance of item calibration. The Wright maps indicate positively skewed person measures and large gaps between H and S items and P and G items.

Additional file 2: Table S1 presents item calibration for the PHuSeG scale showing similar calibration between pre- and posttest. S15 and P8 was rated more difficult after watching movies; while P1 was rated as easier after watching movies. All items showed fit statistics ranging from 0.55 to 1.49. No significant DIF was observed.

The person reliabilities of the scale for pre-posttest were 0.82 and 0.81, while item reliabilities of the scale for pre-posttest were 0.99 and 0.98. PCA confirmed that all items composing the construct of positive character strength did not violate the assumption of unidimensionality.

**Figure 2:**
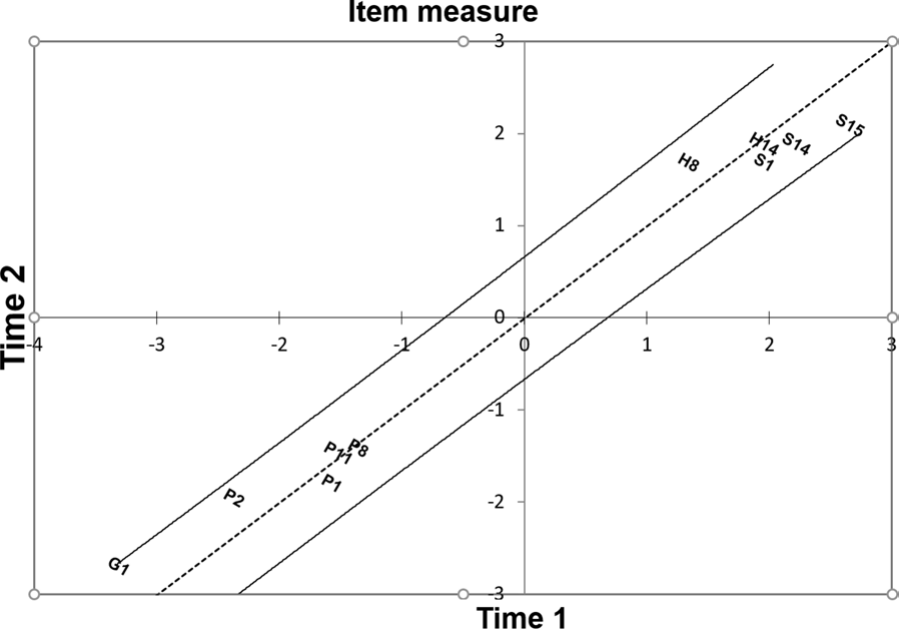
Scatterplot of item difficulty estimates from racked analysis. The line (y = x) indicating no change in item difficulty estimates has been drawn to depict change in item difficulty estimates over time. Items above the line represent items estimated as more difficult by the postintervention assessment while items below the line represent items estimated as less difficult by the postintervention assessment. The centered line is the identity line added for reference. The parallel-identity lines are approximate 95% two-sided confidence bands. Item G1 was higher at Time 2 than at Time 1 (*t* (78)  = − 1.77, p = 0.080), whereas Item S15 was lower (*t* (78)  = 1.27, p = 0.207).

Figure 2 shows that all ten items functioned the same way at both times. G1, S15 and H8 fall on the border of the CI. However, no significant difference was observed in the calibration, and all items were invariant between the two times.

**Figure 3:**
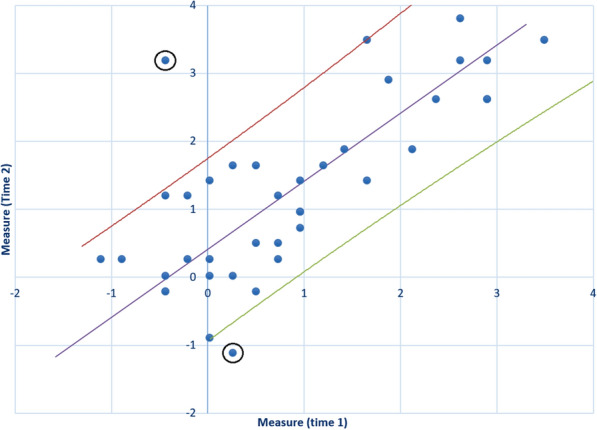
Rasch analysis run on stacked data from the two time points. The person (student) measures at Time 2 are plotted against those at Time 1. Greater measures indicate more positive strength students. Each dot represents one person. Circled dots indicate statistically significant change at time 2 from time 1. The centered line is the identity line added for reference. The parallel-identity lines are approximate 95% two-sided confidence bands. One person (above the lines) scored significantly higher at Time 2 than at Time 1 (*t* (78)  = − 4.45, p < 0.0001), whereas the other person (below the lines) significantly scored lower (*t* (78)  = 2.76, p = 0.007).

Figure 3 shows that seven persons did not change in PHuSeG score (dots along dashed line), 14 gained higher scores after watching the movies (dots on the left side of the centered dash line), while 19 tended to score lower at posttest (dots on the right side of the centered dash line). Only two scored significantly higher at posttest (t = − 4.45 = p < 0.0001, while 1 scored significantly lower on posttest (t = 2.76, p < 0.01). All the rest showed nonsignificant change.

## Discussion

The study aimed to demonstrate how using Rasch model analysis could be applied to a real-life situation using a pre-post design. The overall results showed that Rasch analysis benefitted this type of design. As previously found, interval measures, produced by Rasch analysis are defined by measurement units that are invariant over the entire domain so that the measurement of change is more accurate [29–31]. Because no DIF between pre- and posttest was evident, these changes, if significant, could be affected by the intervention provided.

Concerning the individual level, 37 individuals showed nonsignificant change; only one improved and one worsened significantly. Rasch models solve current shortcomings when assessing change. A measure is determined for each person so that the change can be measured at the individual level. The statistical significance of change is analyzed by means of the standard errors that define the measures.

As documented in related research, the Rasch model is well-developed and used in the field of educational sciences [32–34]. Our study has supported using Rasch analysis for educators to promote growth of character strengths. By that, investigating students who changed might help identify specific characteristics of students linked to positive responses. These factors can then be used to identify those students who are good candidates for the intervention in advance. Second, the intervention does not automatically have to be the same for all students, but can vary.

Rasch analysis showed that all items of the PHuSeG scale were fitted to the model, and were stable over time. However, the skewness of data generally reduced reliability, and the reliability was expected to increase a little in the sample with normal distribution. The large gap shown on the Wright Map also provided us important information that new items of appropriate difficulty should be added.

However, item stability could be questioned due to small sample sizes, and further investigation is warranted. In addition, it seems that the PHuSeG is not sufficiently sensitive to detect the change. PHuSeG was notably derived from the 50 items of the four scales. More items that have responsiveness to change should be identified and included in the new PHuSeG in a further investigation.

Regarding the intervention, using various films did not sufficiently affect the targeted constructs. Movies reflecting a specific construct may be more preferable. In addition to watching movies, other interventions might make the change more evident, e.g., a training to strengthen a specific characteristic might be more suitable.

## Conclusion

Rasch analyses provide more useful information than other measures. Rasch models allow us to examine the invariance of the instrument across time points, and also provides some insight regarding individual data. In addition, it helps validate the psychometric property of the instrument used as well.

## Limitations

This study was conducted using a small sample size and may not be generalizable to all medical students as only first year medical students were selected.

## Supplementary Information

**Additional file 1: Figure S1.** Flowchart of the study.

**Additional file 2: Table S1.** PHuSeG items calibrated at pretest and posttest with mean-square variance–ratio fit statistics.

## Data Availability

The datasets used and/or analyzed during the current study are available from the corresponding author upon reasonable request.

## Abbreviations

INFIT: Information-weighted fit statistics mean square
MNSQ: Mean square
OUTFIT: Outlier-sensitive fit statistics mean square
PCA: Principal Component Analysis
PHuSeG: Prudence, Humility, Self-regulation, and Gratitude
