# Supplementary material for: Changes in character strengths after watching movies: when to use rasch analysis
Source: BMC Res Notes. 2021 Jan 6;14:5. doi: 10.1186/s13104-020-05424-4 (PMC7787118; doi:10.1186/s13104-020-05424-4)
Supplement: Supplementary file 1 — Additional file 1: Figure S1. Flowchart of the study. [file 13104_2020_5424_MOESM2_ESM.docx]

| Table S1. PHuSeG items calibrated at pretest and posttest with mean-square variance –ratio fit statistics | | | | | | | | | |
| --- | --- | --- | --- | --- | --- | --- | --- | --- | --- |
|  |  | Calibration (logit (SE)) | |  | | Fit statistics | | | |
| Item | Statement |  |  | DIF | | IN.MSQ | | OUT.MSQ | |
| label |  | Pretest | Posttest | DIF contrast | p-value | Pretest | Posttest | Pretest | Posttest |
| S1 | Usually I will monitor the progress of my work to reach the goal. | 1.95(.26) | 1.73(.27) | 0.27 | 0.4619 | 0.93 | 0.58 | 0.96 | 0.61 |
| S14 | When I have a goal, I am able to make plans to achieve that goal. | 2.20(.25) | 1.95(.27) | 0.33 | 0.3663 | 0.6 | 0.89 | 0.55 | 0.9 |
| S15 | When I have decided to change something, I will pay full attention to what to do. | 2.63(.24) | 2.17(.27) | 0.57 | 0.1166 | 0.74 | 0.67 | 0.72 | 0.67 |
| H8 | I can accept and appreciate the good aspects of others. | 1.35(.26) | 1.73(.27) | -0.34 | 0.356 | 0.77 | 1.17 | 0.77 | 1.16 |
| H14 | I tend to treat others politely. | 1.95(.26) | 1.95(.27) | 0.07 | 0.8529 | 1.2 | 0.68 | 1.22 | 0.71 |
| G1 | I am grateful for so many things in my life. | -3.34(.25) | -2.73(.24) | -0.62 | 0.0768 | 1.49 | 1.24 | 1.44 | 1.31 |
| P1 | I always think of the consequences before taking action. | -1.49(.23) | -1.83(.23) | 0.22 | 0.5018 | 0.78 | 0.83 | 0.8 | 0.87 |
| P2 | I think that tolerance for better results in the future is good and should be practiced. | -2.37(.23) | -2.00(.23) | -0.41 | 0.2139 | 0.83 | 0.94 | 0.82 | 0.96 |
| P8 | I always have plans for my future. | -1.33(.24) | -1.46(.23) | 0.06 | 0.8609 | 1.32 | 1.13 | 1.27 | 1.06 |
| P11 | I like to ponder decisions by weighing the good and bad effects before making any decisions | -1.49(.23) | -1.51(.23) | -0.05 | 0.8793 | 0.84 | 1.28 | 0.8 | 1.24 |
| OUTFIT.MnSq = outlier-sensitive fit statistics mean square; INFIT.MnSq = information-weighted fit statistics mean square, SE = standard error, DIF = differential item functioning | | | | | | | | | |
