# Supplementary figures and images for: Changes in character strengths after watching movies: when to use rasch analysis
Source: BMC Res Notes. 2021 Jan 6;14:5. doi: 10.1186/s13104-020-05424-4 (PMC7787118; doi:10.1186/s13104-020-05424-4)

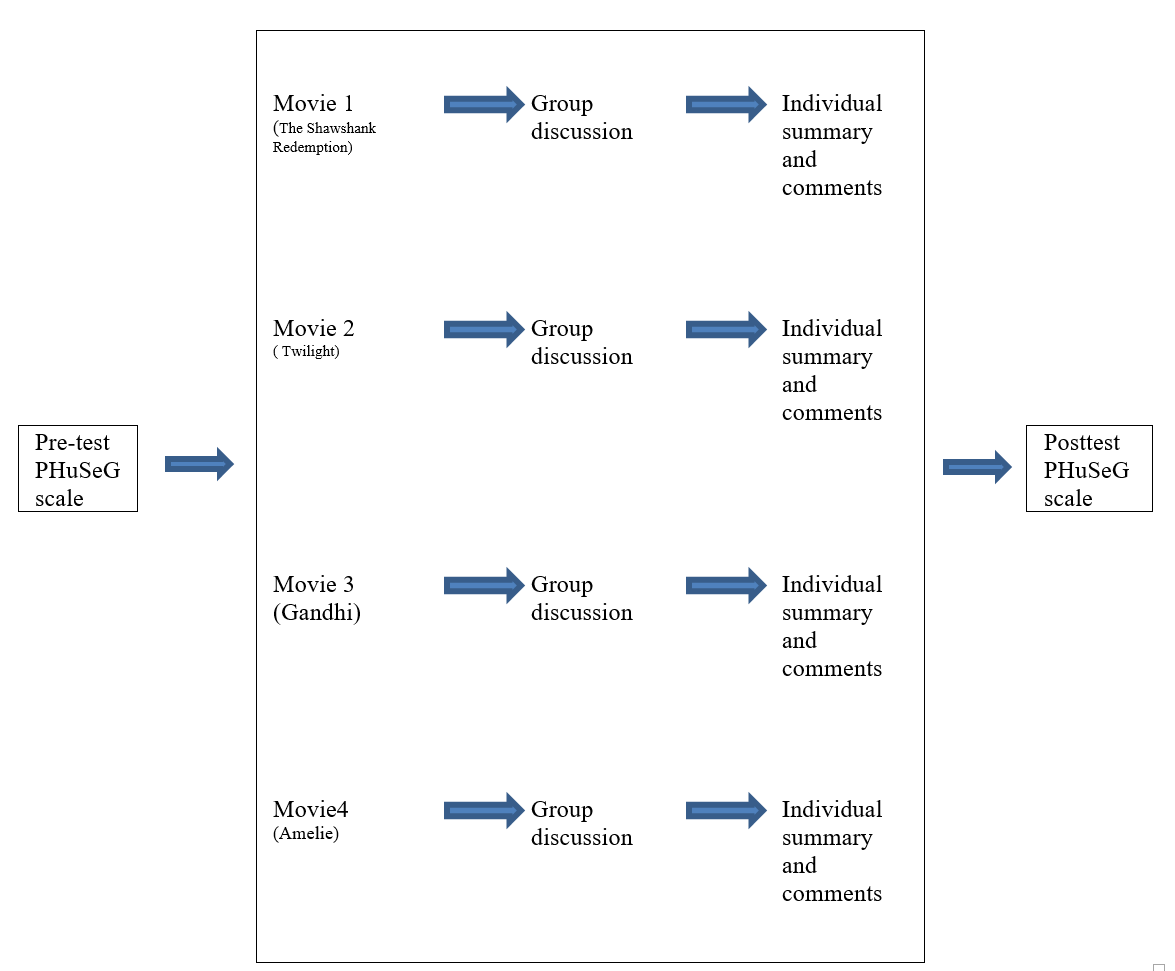

Supplement: Supplementary file 2 — Additional file 2: Table S1. PHuSeG items calibrated at pretest and posttest with mean-square variance–ratio fit statistics. [file 13104_2020_5424_MOESM1_ESM.tif]
